# Apple Pomace as an Ingredient Enriching Wheat Pasta with Health-Promoting Compounds

**DOI:** 10.3390/foods12040804

**Published:** 2023-02-13

**Authors:** Dorota Gumul, Marek Kruczek, Eva Ivanišová, Jacek Słupski, Stanisław Kowalski

**Affiliations:** 1 Department of Carbohydrates Technology and Cereal Processing, Faculty of Food Technology, University of Agriculture in Krakow, Balicka Street 122, 31-149 Krakow, Poland; 2 Institute of Food Science, Faculty of Biotechnology and Food Sciences, Slovak University of Agriculture, Trieda A. Hlinku 2, 949 76 Nitra, Slovakia; 3 Department of Plant Products Technology and Hygiene Nutrition, Faculty of Food Technology, University of Agriculture in Krakow, Balicka Street 122, 31-149 Krakow, Poland

**Keywords:** apple pomace, bioactive compounds, cooking properties, dietary fibre, pasta, polyphenol compounds, texture, dietary fibre

## Abstract

The global overproduction of apples is associated with large amounts of post-production waste, for which new forms of utilization should be sought. Therefore, we aimed to enrich wheat pasta with apple pomace in various percentages (10, 20, 30 and 50%). The content of total polyphenols, individual polyphenols (using UPLC-PDA-MS/MS methods) and dietary fibre, chemical composition and physical properties of the resulting pasta were determined. The addition of apple pomace to pasta resulted in increased levels of pro-health compounds: total polyphenols, phenolic acids, quercetin derivatives, flavon-3-ols and dihydrochalcones as well as dietary fibre. Decreases in hardness and maximum cutting energy were also observed in pasta supplemented with apple pomace compared to control pasta. Water absorption capacity was not influenced by the addition of apple pomace, with the exception of pasta made with 50% apple pomace.

## 1. Introduction

The global annual production of apples reached more than 93 million tons in 2021. The main producers of apples worldwide are China, India, the USA, Turkey and Poland. Poland is the largest producer of apples in the European Union. About 30% of apples are processed into products such as juice, cider and dried products. These technological processes generate up to 30% waste, i.e., apple pomace [1]. It is estimated that plant raw materials such as vegetables and fruits are responsible for more than 20% of losses in the supply chain, becoming bio-waste [2]. According to numerous authors [3,4], about 12% of apple pomace in Poland goes to landfills, which results in its contamination, while the rest is used as a raw material, mainly for the production of pectin, compost for soil fertilization or as a compound feed for animals. Apple pomace can also be applied as a raw material for the production of biogas, ethyl alcohol and organic acids, including citric acid produced by *Aspergillus niger* or as fibre preparations [5,6,7,8]. 

Apple pomace is a heterogeneous mass consisting of skin and flesh (95%), seeds (2–4%) and stems (1%) [9]. It contains numerous compounds, the quantity and quality of which depend on the variety of apples, climatic and soil conditions, the type of apple processing technology and the method of obtaining pomace [10]. However, apple pomace is a valuable source of health-promoting compounds, mainly dietary fibre (DF) (35–65 g/100 g dry matter DM) and bioactive substances from the group of polyphenols (262–856 mg/100 g DM) [11,12]. 

Such compounds present in apple pomace have demonstrated, among other properties, hypoglycaemic, hypocholesterolaemic and anti-cancerogenic activity. They reduce postprandial glucose levels and hypertension, have anti-inflammatory, antiviral, antibacterial, antiallergic and anticoagulant effects and also reduce the risk of such diseases as atherosclerosis and other cardiovascular diseases, cataract, diabetes, genetic damage, bone degeneration and neurodegenerative diseases including Alzheimer’s disease [13,14,15,16,17,18,19].

It was only in the last decade that a number of publications suggested the possibility of enriching traditional products by adding by-products. Fruit and vegetable pomaces in particular have been used to enrich products. Among fruit pomaces, apple pomace deserves attention due to the global overproduction of apples [1,2]. Research on apple pomace concerned their use as a fortifying agent in the production technology of biscuits and muffins [20], where 10% and 15% of wheat flour was replaced with apple pomace. Apple pomace in the amount of 3 to 9% was used to produce gluten-free brown rice crackers [21]. Kirbas et al. [22] replaced 5 to 15% of rice flour with apple pomace flour to make batter and cakes. Meanwhile, Drozdz et al. [23] and Reis et al. [24] obtained extruded snacks with 10 to 30% apple pomace.

Pasta is a traditional cereal product which is readily accepted by consumers, because of its ease of preparation and sensory values [25,26]. An additional asset of pasta is the versatility of its use. A valuable feature of pasta is its low cost of production, making it easily accessible to every household, regardless of their budget. It should be emphasized that the nutritional value of pasta will vary depending on the recipe. Egg pasta will have a higher protein content than pasta based on semolina and water alone, and the amount of dietary fibre depends mainly on its content in semolina [25,26]. Studies on increasing the nutritional and, above all, health-promoting value of pasta have been reported, but it should be noted that the addition of gluten-free ingredients to pasta dough contributes to the weakening of the gluten network and thus the structure of the product [25,26]. This can adversely affect the quality, and above all, the physical characteristics of the pasta; alternatively, it may improve the quality of the product when the ingredients are properly selected, especially those with which the product will be fortified [25,26,27]. Recently, publications on the possibility of producing pasta using fruit or vegetable pomace appeared. However, they mainly focused on the physical characteristics of products, while paying less attention to the health-promoting qualities of this popular product [27,28,29,30,31,32,33]. Therefore, suggesting that apple pomace is a source of health-promoting compounds, we decided to comprehensively investigate pasta with apple pomace, both in terms of the content of nutrients and health-promoting ingredients and the physical characteristics. In this study, we produced, via the process of low-temperature extrusion, egg-wheat pasta fortified with apple pomace. It enabled the utilization of a by-product, and the resulting pasta was enriched with polyphenol compounds (such as quercetin derivatives, chlorogenic acid and phloridzin) present in the apple pomace. The aim of the study was to determine the influence of the addition of different amounts of dried apple pomace (replacing 10, 20, 30 and 50% of wheat flour in recipe) on the content of health-promoting compounds from the polyphenol group, as well as on the texture, quality and functional features of wheat pasta.

## 2. Materials and Methods

### 2.1. Materials

Materials in this work were dried and milled apple pomace (ZPOW Hortino Leżajsk Sp.z.o.o Poland) and egg-wheat pasta prepared with dried apple pomace replacing flour in various percentages: 10, 20, 30 and 50%; see Table 1. Samples were coded as follows:

Control—control wheat pasta (no dried apple pomace added).

P 10%—wheat pasta with 10% apple pomace.

P 20%—wheat pasta with 20% apple pomace.

P 30%—wheat pasta with 30% apple pomace.

P 50%—wheat pasta with 50% apple pomace.

### 2.2. Pasta Preparation

Wheat pasta with dried apple pomace (10, 20, 30 and 50%) and a control sample were obtained by mixing the ingredients according to the recipe (Table 1) in a rotary-roller mixer (Laboratory Spiral Mixer SP 12, Diosna, Germany) for about 20 min at low speed. Pasta production was carried out using a Gina low pressure extruder (Ostoni, Italy). The length of the screw (diameter of 5.5 cm) was 30 cm, and it ended with a forming nozzle (diameter: 1.7 mm). The conditions applied during extrusion were as follows: pressure was about 3.4 × 10^5^ Pa, and temperature was 50 °C. The extruded pasta was dried in a drying chamber (8 h at 40 °C) with up to 12.5% moisture.

About 110 g of prepared pasta was boiled in 1000 mL of distilled water for 8 min. The cooking time has been previously determined in order to obtain *al dente* pasta according to Hirawan et al. [34]. After cooling, the pasta was frozen (−20 °C) and then freeze-dried for 24 h in a freeze dryer (Labconco FreeZone 6, USA) at a temperature of −47 °C and pressure of 37 Pa. The freeze-dried pasta was stored at room temperature for further analysis. Before analysis, the pasta was ground into a powder using a Laboratory Mill 3100 (Perten Instruments, Springfield, IL, USA) equipped with a 0.88 mm mesh.

### 2.3. Chemical Composition of Apple Pomace and Pasta

Protein (Nx5.7) was determined using the Kjeldahl method (AOAC method No. 920.87) using the Kjeltec 2200 extraction unit (Foss, Hillerød, Denmark), fat according to the Soxhlet method (AOAC method No. 953.38) using Soxtec Avanti 2055 (Foss, Denmark), and contents of ash and reducing sugars were determined according to AOAC (2006) methods (AOAC method: 920.183 and AOAC method No. 930.05) [35]. The contents of non-starch polysaccharides, i.e., total (TDF), soluble (SDF) and insoluble (IDF) dietary fibre, were determined using method 32-07 AACCI. TDF was calculated as the sum of soluble and insoluble fractions. Ground samples were dispersed in water and treated with alpha-amylase, protease and glucosidase to remove starch and protein. The residue was precipitated with ethanol, filtered and washed with ethanol and acetone, and dried. TDF was calculated as the mass of the residue minus the protein and ash content [36]. Pectin concentration was determined using the carbazole method. Briefly, 2 g of pomace was weighed, 40 mL of ethanol (80%) was added, and then they were heated under reflux (30 min) and filtered. The filter with the precipitate was transferred to a flask, 50 mL of distilled water was added, brought to the boil, filtered hot and the filtrate was made up to 100 mL. The resulting extract contained pectins [37]. Each of the above-mentioned determinations was performed in at least 2 replicates.

### 2.4. Content of Antioxidant in Apple Pomace and Pasta

Determination of bioactive compounds was performed using spectrophotometric methods. Antioxidant constituents were determined in the ethanol extracts; 0.6 g of the sample was dissolved in 30 mL of 80 g/100 g ethanol, shaken in the dark for 120 min (electric shaker: type WB22, Memmert, Schwabach, Germany), and centrifuged (15 min, 4500 rpm. 1050× *g*) in a centrifuge (type MPW-350, MPW MED. Instruments, Warsaw, Poland). The supernatant was decanted and stored at −20 °C for further analyses. 

Determination of total polyphenol content (TPC) was performed with the spectrophotometric method using Folin–Ciocalteu reagent (F–C reagent), in accordance with Singleton et al. [38]; 5 mL of the extract was diluted to a volume of 50 mL with distilled water, and 5 mL of the diluted extract was combined with 0.25 mL of F–C reagent (previously diluted with distilled water in the proportion 1:1 *v/v*) and 0.5 mL of 7% Na_2_CO_3_. The contents were vortexed (WF2, Janke & Kunkel, Staufen, Germany) and stored for 30 min in a dark place. The absorbance was measured using a Helios Gamma 100–240 (Thermo Fisher Scientific, Runcorn, UK), at the wavelength λ = 760 nm. The results were converted to mg catechin/100 g DM. 

Determination of flavonoids was performed using the spectrophotometric method, in accordance with El Hariri et al. [39]; 0.5 mL of the extract was combined with 1.8 mL of distilled water and 0.2 mL of 2-aminoethyldiphenylborate reagent in a test tube. The contents were vortexed, and the absorbance was measured at the wavelength λ = 404 nm. Flavonoid content was expressed as mg of rutin/100 g DM. 

Determination of polyphenols individual was performed using ultra-performance liquid chromatography/photodiode array detection/tandem mass spectrometry (UPLC-PDA-MS/MS). Samples of the raw material (about 1 g) were extracted with 10 mL of a mixture containing methanol of HPLC purity level (30 mL/100 mL), ascorbic acid (2.0 g/100 mL) and acetic acid in the amount of 1.0 mL/100 mL of the reagent. Extraction was carried out twice by incubation for 20 min under sonication (Sonic 6D, Polsonic, Warsaw, Poland) and occasionally mixing. The suspension was then centrifuged at 19,000× *g* for 10 min and the supernatant was filtered through a 0.20 μm Hydrophilic PTFE membrane (marble filter Simplicity Millex, Merck, Darmstadt, Germany) and used for analysis. Phenolic compounds were analysed using an Acquity Ultra-Performance Liquid Chromatograph equipped with a Binary Solvent Manager (BSM), Sample Manager (SM) combined with a PDA detector and quadrilateral time of flight (Q-TOF) (Waters, Manchester, United Kingdom). The analysis was carried out on a 2.1 × 100 mm UPLC BEH C18 column containing 1.7 μm particles (Waters, Manchester, UK). Data were collected and analysed using MassLynx v 4.1 (Waters) software. Anthocyanins were analysed in the positive ion mode and the remaining polyphenols in the negative ion mode. Quantification of phenolic compounds was performed using external standard curves, using reference compounds selected on the basis of the target analyte/structure standard (chemical structure or functional group). The standards were prepared in concentrations ranging from 0.05 to 5 mg/mL. The resulting correlation coefficient was R^2^ ≤ 0.9998. The results were expressed in mg per 100 g DM.

### 2.5. Texture Profile Analysis: Determination of Maximum Cutting Force and Energy

The maximum cutting force and energy for freshly cooked pasta were determined on a TAXT2 plus texture analyser (Stable Micro Systems, Godalming, UK) using a Warner-Bratzle adapter with a flat knife for cutting the sample at a speed of 3 mm/s. Exponent v. 4.0.13.0 software was used to collect data. The measurements were made in seven replications; two extreme results were discarded, and the others were used to calculate the arithmetic mean.

### 2.6. Water Absorption of Pasta

The water absorption of pasta was determined in accordance with the methodology of Tudoric et al. [40]. Briefly, dry pasta (10 g) was weighed, cooked in 500 mL of water, drained and then reweighed. The measurement was repeated three times. 

Calculation of the water absorption (WA) of the pasta (%) was made on the basis of the formula:

WA = (a − b)/a,(1)

where:

a—is the weight of cooked pasta (g);

b—is the weight of pasta before cooking (g) [40].

### 2.7. Statistical Analysis

The experimental data were subjected to analysis of variance (Duncan’s test), at the confidence level of 0.05, using Statistica v. 8.0 (StatSoft, Inc., Tulsa, OK, USA). All measurements were made at least in duplicate. Pearson’s correlation coefficient was calculated at alpha = 0.01.

## 3. Results and Discussion

### 3.1. Apple Pomace Characteristics

**Figure 1:**
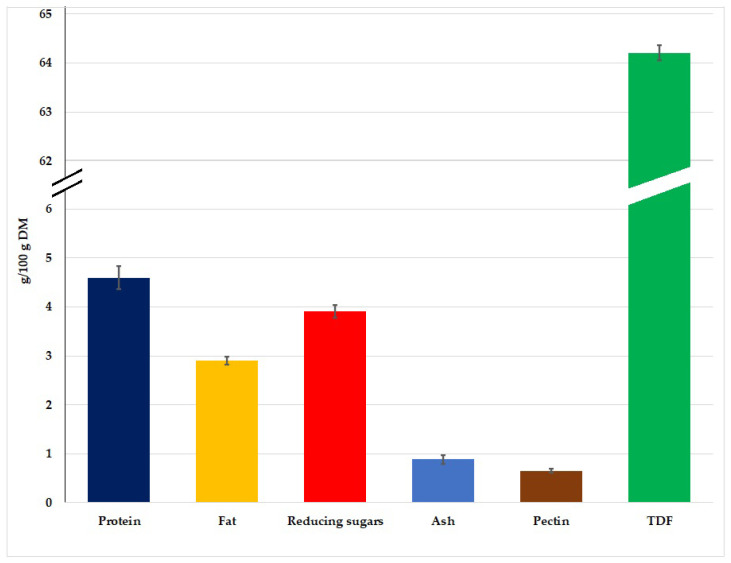
Basic chemical composition of apple pomace.

The chemical composition of apple pomace is presented in Figure 1. According to Wang et al. [41], the amount of protein was 3.8 g/100 g DM, fat was 3.8 g/100 g DM, TDF was about 26.5 g/100 g DM, and ash was 1.8 g/100 g DM in apple pomace. Jin et al. [42] determined the amount of protein, fat and ash at the levels of 4.7, 4.2 and 1.5 g/100 g DM. Jannati et al. [43] determined the amounts of protein, fat, TDF and ash as 1.2, 0.6, 14.5 and 2.5 g/100 g DM, respectively. Ktenioudaki et al. [44] reported that the amounts of protein, fat, TDF and ash were 2.4, 2.7, 42.5 and 1.7 g/100 g DM. According to Pieszka et al. [45] and Leyva-Corral et al. [46], the amounts of protein, fat, TDF and ash were, respectively, 3.73–3.8 g/100 g DM, 1.8–2 g/100 g DM, 36–45 g/100 g DM and 1.88–2 g/100 g DM. 

This study showed that the constituent with the largest share in the dry matter of apple pomace is fibre, which was also confirmed by the above-mentioned authors [41,43,45,46]. It should also be emphasized that the results of our research were consistent with the results of the above authors, and the few discrepancies may result from climatic, soil, agrotechnical conditions or apple variety and the method of obtaining pomace [45]. In our study, it is important that we had a mixture without varieties of apple pomace from various varieties of apples grown in eastern Poland and the method of obtaining the pomace (methods of press and number of press cycles). According to Constenla et al. [10] and Kieliszek et al. [47], the above-mentioned factors may affect the composition of pomace, because the amount of protein may be in the range of 2.7–5.7 g/100 g DM, ash 1.1–2.2 g/100 g DM, carbohydrate 20–57.4 g/110 g DM and dietary fibre 43–61.6 g/100 g DM.

It should be noted that the dietary fibre (DF) of apple pomace constitutes two-thirds of the insoluble fraction, which consists mainly of cellulose, hemicellulose and lignin, and pectins are the dominant component of the soluble fibre fraction [48]. The amount of pectins recorded in this work in apple pomace is 0.65 g/100 g DM. Pectins are very important compounds because of their various physiological properties. They exert prebiotic effects and are also fermented in the large intestine by the local microflora, resulting in the formation of short-chain fatty acids (SCFA) which are absorbed and converted into colonic mucosa, liver or peripheral tissues. A relationship has been identified between the consumption of pectins and maintenance of regular blood cholesterol concentrations, and the reduction of post-prandial glycemic responses [48].

Apple pomace is a valuable source of polyphenols and flavonoids, the amounts of which determined in this research were 89.39 mg gallic acid/100 g DM and 94.27 mg rutin/100 g DM. According to the study by Ćetković et al. [11], the total amount of polyphenols ranged in apple pomace from 420 to 867 mg chlorogenic acid/100 g DM, whereas flavonoids in apple pomace ranged from 45 to 119 mg rutin/100 g DMDM [11]. Persic et al. [49] noted the total polyphenol content in the range of 19–50 mg gallic acid/100 g DM [49]. The content of total polyphenols in plant material is influenced not only by the extraction conditions, but also by the various ways in which the results were expressed (e.g., another type of phenolic compound used to calculate polyphenols) [50].

**Figure 2:**
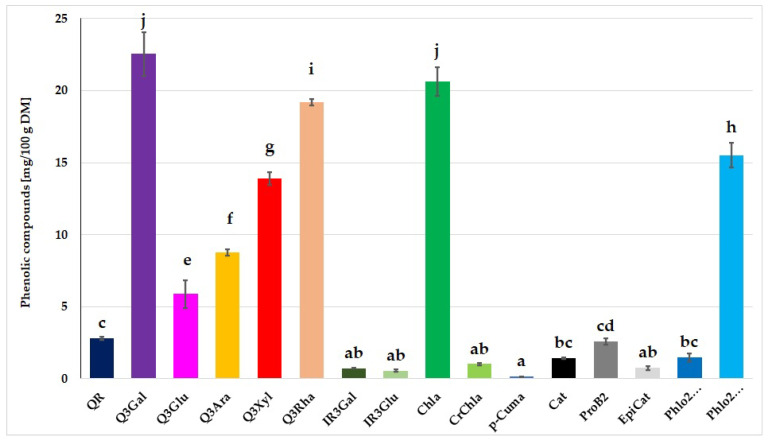
Quality and quantity of phenolic compounds in apple pomace: QR (quercetin-O-rutinoside), Q3Gal (quercetin-3-O-galactoside), Q3Glu (quercetin-3-O-glucoside), Q3Ara (quercetin-3-O-arabinoside), Q3Xyl (quercetin-3-O-xyloside), Q3Rha (quercetin-3-O-rhamnoside), IR3Gal (isorhamnetin-3-O-galactoside), IR3Glu (isorhamnetin-3-O-glucoside), Chla (chlorogenic acid), CrChla (cryptochlorogenic acid), p-Cuma (p-coumaroylquinic acid), Cat ((+) catechin), ProB2 (procyanidin B2), EpiCat ((-) epicatechin), Phlo2Xyl (phloretin-2-O-xylosylglucoside), Phlo2Gluc (phloretin 2-O-glucoside (phloridzin)). Different letters over the bars represent the statistically significant difference of average values (α = 0.05).

UPLC-PDA-MS/MS analysis of the profile of individual phenolic compounds present in apple pomace was performed (Figure 2). It was found that among phenolic acids, chlorogenic acid had the largest share (20.65 mg/100 g DM Figure 2), which is similar to the other authors’ results of 92–104 mg/100 g DM [51]. Other phenolic acids were also identified; cryptochlorogenic acid was determined at 1.03 mg/100 g DM and p-coumaroylquinic acid at 0.16 mg/100 g DM (Figure 2). According to Kammerer et al. [12], the amount of p-coumaroylquinic acid was 0.18 mg/100 g DM [12]. Among the analysed flavonols, quercetin derivatives had a large proportion, while quercetin-3-O-galactoside, quercetin-3-O-rhamnoside and quercetin-3-O-xyloside were dominant (Figure 2). Other authors have also noted similar results for quercetin-3-O-glucoside: 28.6–61.0 mg/100 g DM [11] and 52.1–68.1 mg/100 g DM [52]. Flavan-3-ols and dihydrochalcones are also a very important group of phenolic compounds in apple pomace. Among flavan-3-ols, catechin at 1.40 mg/100 g DM, procyanidin B2 at 2.53 mg/100 g DM and epicatechin at 0.70 mg/100 g DM were noted (Figure 2). The catechin content was previously reported to be 1.7–12.7 mg/100 g DM [11], 0.24 mg/100 g DM [12] and 0.94–1.4 mg/100 g DM [52]. The content of epicatechin in the apple pomace assessed by other authors was: 2.4–17.3 mg/100 g DM [11]; 0.93 mg/100 g DM [12]; 14–19 mg/100 g DM [52]; 12.23 mg/100 g DM [46]. They also determined the amount of procyanidin B2: 2.3–10 mg/100 g DM [51], 0.93 mg/100 g DM [12] and 9.3–16 mg/100 g DM [52]. Among dihydrochalcones in apple pomace, phloridzin was dominant, which was determined at 15.47 mg/100 g DM (Figure 2). Similar values (17.97 mg/100 g DM) were reported by Leyva-Corral et al. [46]. In the study by Lyu et al. [9], the content of dihydrochalcones in apple pomace ranged from 68.8 to 253.5 mg/100 g DM.

### 3.2. Characteristics of Pasta with Different Percentage Content of Apple Pomace

Table 2 contains the results of the determination of total polyphenols, flavonoids, proteins, fat, reducing sugars and ash in cooked pasta with or without apple pomace. The control sample contained 11.44 g/100 g DM protein, and the addition of apple pomace reduced the amount of this ingredient from 6% to 19% compared to the control (Table 2). The partial replacement of wheat flour (which is a source of protein) with apple pomace and also the cooking process probably resulted in a dilution effect of this important nutrient [53].

In the case of fat, the highest content was observed in the control sample (2.25 g/100 g DM), and with increasing apple pomace content, the amount of fat decreased in the range from 9% to 38% as compared to the control. Pasta with the highest percentage of apple pomace contained the lowest amount of fat (1.39 g/100 g DM; Table 2). The main source of fat in the pasta was egg mass, the addition of which was the same in all samples (Table 1 and Table 2). The addition of eggs improves the nutritional value of the obtained pasta. Although apple pomace contains more fat (2.90 g/100 g DM) than wheat flour (about 1.0 g/100 g DM), which was substituted, it was likely that gluten present in wheat flour bound egg fat better than apple pomace, which was why during the cooking process fat could be partially washed out [25]. According to available sources, the degree of fat binding depends mostly on the physicochemical properties of the raw material and next on the production process parameters [54], so it could be the reason for the reduced fat content in the final product. It was found that, regardless of the proportion of applied apple pomace, the content of reducing sugars in pasta was at the same level (about 1.07 g/100 g DM) and about 27% higher than the control. Although the source of reduced sugars in pasta was apple pomace, the process of cooking the pasta probably contributed to the washing out of the above ingredients from the products. Only strongly bound reducing sugars remained, which resulted in similar results, mostly not correlated with the applied proportion of apple pomace. However, the content of mineral components ranged from 0.50 g/100 g DM to 0.81 g/100 g DM, and the largest amount was observed in pasta with 50% apple pomace addition (0.81 g/100 g DM; Table 2). It is likely that the cooking of pasta contributed to a partial leaching of the above-mentioned ingredients from products, which resulted in different results that were not especially correlated with the applied apple pomace.

In a study by Gałkowska et al. [28] on noodles with the addition of 5 and 10% blackcurrant pomace, the amount of protein in these noodles did not change compared to the control. In contrast, the fat content increased twofold and the ash content by about 40% in pasta supplemented with blackcurrant when compared to the control [28]. In the study by Nur Azura et al. [31] on yellow alkaline noodles with mango peel (10–30%), it was noted that the content of ash, fat and protein in the noodles did not change after the use of the above-mentioned additive compared to the control. The amount of crude fibre increased 2–14-fold, and the amount of carbohydrate decreased by 5–25% after the introduction of mango peel to pasta relative to the control. In research by Isa et al. [32], in which pasta with mango powder (2–6%) was obtained, it was found that the ash content increased by an average of 33%, protein by 3% and dietary fibre 7-fold compared to the control. The amount of fat decreased by 50% in the mango peel powder pasta compared to the control.

Total polyphenol and flavonoid contents in pasta with the addition of apple pomace is given in Table 2. It was found that the total amount of polyphenols (TPC) and flavonoids, after apple pomace addition, increased from 120% to 410% and from 90% to 774% as compared to the control. It was also found that this increase was proportional to the level of pomace added, and the largest increase was observed for 50% replacement of wheat flour by apple pomace (Table 2).

Ajila et al. [55] reported a 3.9-fold increase in the total polyphenol content in wheat pasta with mango peel powder (MPP) in the range up to 20% when compared to the control [55]. In the study by Tolve et al. [29] concerning the influence of the addition of grape pomace on the quality of durum wheat pasta, it was observed that the amount of polyphenols after the use of 5 and 10% grape pomace addition increased, respectively, seven- and twelvefold compared to the control.

Taking into account the profiles of flavonoids, phenolic acids, flavan-3-ols and dihydrochalcones which were identified by the UPLC-PDA-MS/MS method, it was found that the control sample, which consisted mainly of wheat flour, contained only two phenolic compounds (di-p-coumaroylspermidine and feruloylquinic acid), and the addition of apple pomace caused a significant increase in the amount of phenolic compounds (Figure 2, Table 3). The content of phenolic acids increased in pasta with the addition of apple pomace, although this increase was smaller than expected. It was probably related to the production stages (Table 3). It can be suggested that during the low-temperature extrusion process, there was a partial release of phenolic acids from the pomace fibre fraction. Pasta drying could also decarboxylate phenolic acids to 4-vinyl guaiacol, while the cooking process additionally resulted in their degradation, leaching and dissolving them in water [56]. Michalska et al. [57] clearly observed that polyphenol losses were strongly affected by the type and parameters of drying.

Pasta samples with apple pomace were characterized by a significantly higher content of quercetin derivatives as well as flavon-3-ols and dihydrochalcones. It was associated with applied addition because, as already mentioned, apple pomace is an excellent source of the above-mentioned biologically active compounds, whereas the control pasta does not contain such compounds (Table 3). Nevertheless, it should be remembered that the particular stage of pasta production can contribute to the losses of these compounds, similarly to phenolic acids. Nevertheless, apple pomace applied as an additive enriched pasta with antioxidants (Table 3). Ajila et al. [55] reached a similar conclusion when pasta was fortified with additional mango peel [55]. Similar conclusions were made by Tolve et al. [29], who studied the effect of adding grape pomace on the polyphenol content in pasta. Gaita et al. [58], examining the effect of grape pomace (3–9%) on the content of polyphenols, noted that the higher the level of the additive, the higher the polyphenol content in pasta with the above-mentioned additive compared to the control. Gaita et al. [58] observed an increase in polyphenol content in the range of 31–97% in pasta with the addition of 3 to 9% grape pomace compared to the control.

Data related to dietary fibre (DF) in pasta with apple pomace addition are presented in Table 4. It was found that the amount of insoluble and soluble DF increased, respectively, from 3.6- to 17-fold and from 1- to 4.4-fold in pasta made with apple pomace in comparison to the control. At the same time, it was found that this increase was proportional to the increase in the pomace content, and the largest increase was observed for the pasta with 50% apple pomace content. Pasta supplemented with apple pomace was also characterized by a high total fibre content (11–36.73 g/100 g DM; Table 4).

In the research performed by Ajila et al. [55], the effects of the addition of MPP to pasta on the content of soluble and water-insoluble fractions and total DF fraction were evaluated [55]. In pasta with MPP addition (0–20%), an increase in the content of DF fractions was observed as follows: from 24% to 57% (water-soluble fraction), from 87% to 144% (water-insoluble fraction) and from 61% to 107% (total fibre) compared to the control. In the study by Gałkowska et al. [28], the amount of DF increased 2.5- to 5-fold after the application of blackcurrant pomace in pasta relative to the control. Pasta with blackcurrant pomace was characterized by a two-fold increase in the soluble fraction of DF, and the insoluble fraction increased 4- to 7-fold when compared to the control. In research by Padalino et al. [30] on the supplementation of pasta with tomato pomace, they observed that the use of 10 and 15% tomato pomace resulted in the increases in the insoluble DF fraction content from 35 to 60%, soluble fraction from 23 to 43% and total DF from 31 to 54% as compared to the control. In the study by Kultys and Moczkowska-Wyrwisz [33] for pasta with carrot pomace from 10 to 30% and beetroot-apple pomace (10–30%), an increase in total dietary fibre was observed, on average by 85% in pasta with carrot pomace and by 80% in pasta with beetroot-apple pomace compared to the control. The insoluble fraction of DF increased in pasta with carrot pomace in the range of 49 to 108%, and in pasta with beetroot-apple pomace it increased in the range of 73 to 150% compared to the control. The soluble DF fraction was up to three and a half times higher in pasta with carrot pomace, and in beetroot-apple pomace it was twice as high as the control. It can be concluded that the content of DF and its fractions in our study corresponded to the proportion of apple pomace in pasta (Table 4) because apple pomace was a rich source of DF (64.21 g/100 g DM), which is confirmed by the studies of the above-cited authors [28,33,55]. 

In addition to the nutritional and health-promoting properties of pasta, this product should also be characterized by the desired culinary features (especially water absorption) and textural properties (hardness), i.e., the characteristics of pasta that were revealed after hydrothermal treatment (cooking). These properties are primarily determined by the pasta recipe and processing technology. It should be remembered that the introduction of an additional enriching ingredient to the basic pasta recipe can cause some disturbances in the starch-gluten network microstructure and, consequently, can lead to modification of the culinary and sensory properties (including textural) of the finished product [25,33]. 

The hardness and maximum cutting energy of pasta supplemented with apple pomace were lower than those of the control (Table 4). Pasta with the largest percentage of apple pomace (50%) was characterized by the lowest cutting energy. This addition caused a decrease in hardness of the final product by approximately 30% as compared to the control, so these pastas were softer and more susceptible to damage (Table 4). Many factors could have an impact on these results, first of all the starch capacity for pasting, which according to Dexter & Matsuo [59] affected the hardness of cooked pasta. Secondly, DF, which was a main component of apple pomace, had a decisive impact on the hardness of the finished products [55]. However, a lot depends on the structure, form and composition of DF present in the product. For example, inulin (polysaccharide) reduces the hardness of finished pasta [60]; arabinoxylans, which are water soluble, do not affect the texture of cooked pasta [61]; and bran fibre increases the hardness of the pasta [62].

In their research, Ajila et al. [55] found that the increase in hardness from 19% to 67% compared to the control was directly proportional to the proportion of applied MPP [55]. In the studies of Padalino et al. [30] on the effect of tomato pomace on the hardness of pasta, a two-fold increase in hardness after adding this additive compared to the control was noted. A similar increase in hardness was noted by Xu et al. [27] by fortifying the pasta with 5 to 20% apple pomace. In the study by Kultys and Moczkowska-Wyrwisz [33], pasta with 10–20% carrot pomace and beetroot-apple pomace had slightly lower hardness. However, the 30% share of the above-mentioned additives resulted in a 20% decrease in hardness in the case of carrot pomace and 25% in the case of beetroot-apple pomace of these enriched pastas compared to the control. In the research of Nur Azura et al. [31] on noodles with mango peel powder (10–30%), no change in hardness was noted compared to the control. In the case of the research presented here, it should be taken into consideration that during the dough formation, egg albumins formed disulphide bonds (S-S) with gluten proteins, participating in the formation of the gluten network. The fibre that was introduced with the apple pomace interfered with the protein–starch matrix during dough development. Fibre became another ingredient competing for water with proteins and starch. Assuming that fibre had a greater water absorption capacity than starch, it interfered with the transformation of protein structure as well as the incorporation of starch granules into the gluten network [25,26,27]. As a consequence, the structure of the pasta after drying can be less homogeneous and less compact, i.e., less hard, hence the lower observed hardness of the pasta supplemented with apple pomace as compared to the control (Table 4). As previously mentioned, the hardness of the product was affected by the ingredients presented in the recipe. Fruit pomaces were a source of various types of DF with different compositions, molecular weights and degrees of polymerization or methoxylation as in the case of pectins [60], which mainly determined the hardness of the finished product, and this has been confirmed by other authors [27,30,55].

Water absorption is the ability of a product to bind water, in this case during cooking. Pasta with good parameters should be characterized by high water absorption. The water absorption of the pasta is directly proportional to the cooking time of the pasta. The longer the cooking time, the more water is bound in the product [33]. In this study, all samples were prepared according to the established optimal cooking time. The absorption of water by pasta is affected not only by the content of starch and pectins, which have, among others, hydrophilic properties [63], but also the structure of starch, the number and location of hydroxyl groups in pectin and the degree of their methylation. This parameter is influenced by the internal structure of starch and pectins in dried pasta, as well as the presence of polyphenols, as well as the composition of dietary fibre [25,33]. All these factors affected the interactions between the product ingredients and water, and finally the water absorption capacity [64]. 

**Figure 3:**
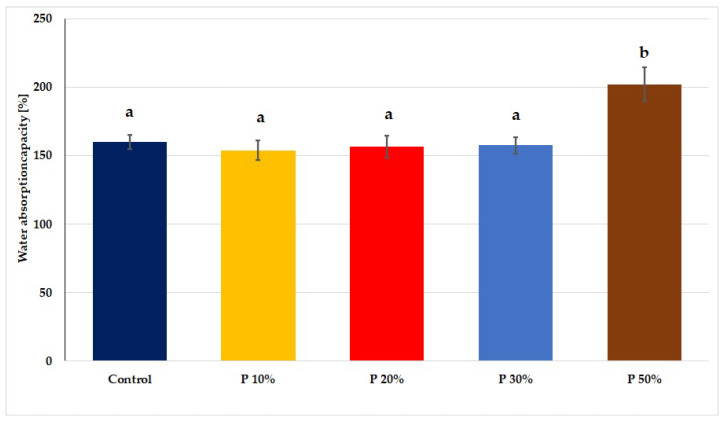
Water absorption capacity of wheat pasta with added apple pomace. Different letters over the bars mean statistically different average values (α = 0.05).

Only the percentage of pomace at the level of 50% increases water absorption by approximately 26% (Figure 3) compared to other samples. It should be emphasized that along with the increase in the percentage of apple pomace, the content of pectins in the finished products also increased. However, the degree of methylation of these pectins was also an important factor, because highly methylated pectins (which include apple pomace pectins [65]) were more hydrophobic than pectin molecules with a low degree of methylation, which could have affected the results [64]. Additionally, as reported by Sivam et al. [66], polyphenols, of which apple pomace is a valuable source, could combine with proteins, starch and other polysaccharides through hydrogen bonds, leading to increased intermolecular interactions, and thus changing their hydrophilic properties, which affected the water absorption of products. Mineral components, which were also provided by apple pomace, increase the water absorption of finished products [67]. This was confirmed by the high level of ash in pasta supplemented with 50% pomace (0.81 g/100 g DM; Table 2), which contributed to, among other effects, up to 26% increase in water adsorption in these final products (Figure 3). Taking into account the recipe of pasta with apple pomace obtained in this research, it should be emphasized that the comparable water absorption of this pasta with the control sample (except for pasta with 50% apple pomace) may be due to the fact that the product was obtained by means of low-temperature extrusion during which free flour fats and egg monoacylglycerols interacted with the starch polymer amylose, thus limiting its swelling.

According to Gałkowska et al. [28], pasta fortified with blackcurrant pomace was characterized by lower water absorption than the control, and the greater the addition of blackcurrant pomace, the lower the water absorption by the pasta. Xu et al. [27] observed in pasta with apple pomace that the increase in the proportion of apple pomace was associated with a slight increase in water absorption by the product (4%) as compared to the control. However, in the study by Tolve et al. [29], in which pasta was fortified with grape pomace, it was found that with the increase in the percentage of this additive, the water absorption of the pasta decreased. The research by Kultys and Moczkowska-Wyrwisz [33] clearly showed that the recipe and the type of additive used influence the water absorption of pasta. In the studies mentioned above, the authors used the same percentage of two additives (10–30%), carrot pomace and beetroot-apple pomace, claiming that the water absorption of pasta increases when carrot pomace is used or does not change when beetroot-apple pomace is used compared to the control. This can be explained by the different composition of dietary fibre in these two types of additives. In carrot pomace, the soluble fraction of fibre predominated, which guaranteed greater water absorption. It can therefore be said that in our study, higher water absorption (by 26%) by pasta with 50% apple pomace compared to the control may be associated not only with a higher content of ash, but also with the highest content of soluble fraction of dietary fibre in this pasta (Table 4).

Sun-Waterhouse et al. [64] observed that the proportion of elderberry juice in wheat pasta reduced its water absorption, except for pasta where there were also highly methylated pectins. In the sample with highly methylated pectins (exceeding hydrophobic properties) and elderberry juice, greater water absorption was observed than in pasta with only highly methylated pectins, most likely due to the presence of polyphenols in the added juice, which changed the conformation of gluten proteins and the structure of starch present in the flour, probably affecting the water absorption of the obtained pasta. Sun-Waterhouse et al. [64] also found that the addition of blackcurrant juice to wheat pasta reduced the volume of cooked pasta compared to samples without such addition.

## 4. Conclusions

It was found that apple pomace enriched wheat pasta with polyphenolic compounds (from 120% to 410% as compared to the control) and afforded an almost 8-fold increase in flavonoid content. Apple pomace also enriched wheat pasta with phenol acids, quercetin derivatives, flavon-3-ols and dihydrochalcones. The addition of apple pomace to pasta resulted in the growth of another health-promoting ingredient, dietary fibre. The content of dietary fibre was three to ten times higher in pasta with apple pomace relative to the control. Pasta with apple pomace was characterized by a higher content of soluble and insoluble fractions of dietary fibre, from 1 to 4.5 and from 3.5 to 17 times higher, respectively, compared to the control. In chemical composition, decreases in protein and fat content were observed, which were directly proportional to the percentage of apple pomace added. An increase in mineral components was also observed, particularly visible for the highest percentage of added pomace. 

The hardness and maximum cutting energy for pasta decreased as the percentage of apple pomace increased in comparison to the reference sample. The addition of apple pomace to pasta did not negatively affect the water absorption of this product. 

It was found that the smallest percentage (10%) of apple pomace in pasta resulted in the enrichment of wheat pasta with health-promoting compounds. This addition of apple pomace provided a 2-fold increase in polyphenols and flavonoids, and a significant increase in quercetin derivatives (quercetin-3-xyloside and quercetin-3-rhamnoside), chlorogenic and phloridzin. Such a small addition also results in a fivefold increase in the insoluble fraction, a twofold increase in the soluble fraction and a threefold increase in total dietary fibre. It also ensures the appropriate hardness and water absorption of pasta, which are important cooking properties.

## Figures and Tables

**Table 1:** Composition of the mixture used to prepare wheat pasta.

	Wheat Flour (g)	Apple Pomace (g)	Distilled Water (mL)	Egg Mass (g)	Salt (g)
Control	500	0	150	56	5
P 10%	450	50	160	56	5
P 20%	400	100	170	56	5
P 30%	350	150	210	56	5
P 50%	250	250	340	56	5

**Table 2:** Total polyphenols, flavonoid content and chemical composition of gluten pasta with apple pomace.

Sample	Total Polyphenols	Flavonoids	Protein	Fat	Reducing Sugars	Ash
	(mg catechin/100 g DM)	(mg rutin/100 g DM)	(g/100 g DM)			
Control	21.87 ± 0.92 a *	10.01 ± 1.76 a	11.44 ± 0.09 e *	2.25 ± 0.05 e	0.84 ± 0.03 a	0.55 ± 0.01 b
P 10%	47.79 ± 0.00 b	19.03 ± 1.46 b	10.78 ± 0.06 d	2.05 ± 0.05 d	1.07 ± 0.01 b	0.50 ± 0.01 a
P 20%	68.03 ± 1.11 c	38.18 ± 1.95 c	10.51 ± 0.12 c	1.92 ± 0.06 c	1.07 ± 0.01 b	0.63 ± 0.01 d
P 30%	89.06 ± 1.59 d	53.11 ± 1.29 d	10.14 ± 0.03 b	1.73 ± 0.01 b	1.07 ± 0.01 b	0.59 ± 0.01 c
P 50%	111.28 ± 0.00 e	87.48 ± 1.46 e	9.23 ± 0.04 a	1.39 ± 0.05 a	1.05 ± 0.01 b	0.81 ± 0.01 e

* Different letters in the column represent the statistically significant difference of average values (α = 0.05).

**Table 3:** Quality and quantity of phenolic compounds in pasta with apple pomace.

Compound	Control	P 10%	P 20%	P 30%	P 50%
Flavonols (mg/100 g DM)					
isorhamnetin-3-O-galactoside	0.00 ± 0.00 a	0.06 ± 0.00 b	0.19 ± 0.00 c	0.34 ± 0.07 d	0.51 ± 0.11 e
isorhamnetin-3-O-glucoside	0.00 ± 0.00 a	0.09 ± 0.00 b	0.27 ± 0.02 c	0.38 ± 0.00 d	0.45 ± 0.09 d
luteolin 6-C-hexoside-O-hexoside	0.00 ± 0.00 a *	0.00 ± 0.00 a	0.00 ± 0.00 a	0.00 ± 0.00 a	0.00 ± 0.00 a
luteolin O- hexoside-C-hexoside	0.00 ± 0.00 a	0.00 ± 0.00 a	0.00 ± 0.00 a	0.00 ± 0.00 a	0.00 ± 0.00 a
quercetin-O-rutinoside	0.00 ± 0.00 a	0.19 ± 0.00 b	0.51 ± 0.20 c	0.73 ± 0.12 d	1.05 ± 0.10 e
quercetin-3-O-galactoside	0.00 ± 0.00 a	1.91 ± 0.15 b	5.22 ± 0.13 c	7.32 ± 0.17 d	8.97 ± 0.25 e
quercetin-3-O-glucoside	0.00 ± 0.00 a	0.17 ± 0.02 b	1.24 ± 0.00 c	2.35 ± 0.09 d	3.41 ± 0.12 e
quercetin-3-O-arabinoside	0.00 ± 0.00 a	0.58 ± 0.11 b	1.70 ± 0.00 c	3.03 ± 0.30 d	4.19 ± 0.13 e
quercetin-3-O-xyloside	0.00 ± 0.00 a	1.91 ± 0.10 b	4.26 ± 0.27 c	5.18 ± 0.23 d	6.28 ± 0.51 e
quercetin-3-O-rhamnoside	0.00 ± 0.00 a	1.90 ± 0.00 b	4.80 ± 0.00 c	5.84 ± 0.57 d	6.72 ± 0.14 e
Phenolic acids (mg/100 g DM)					
chlorogenic acid	0.00 ± 0.00 a	1.23 ± 0.00 b	3.48 ± 0.00 c	4.15 ± 0.09 d	5.27 ± 0.13 e
cryptochlorogenic acid	0.00 ± 0.00 a	0.08 ± 0.00 b	0.19 ± 0.00 c	0.37 ± 0.06 d	0.61 ± 0.05 e
p-coumaroylquinic acid	0.00 ± 0.00 a	0.10 ± 0.11 a	0.26 ± 0.05 ab	0.32 ± 0.01 c	0.45 ± 0.09 d
caffeoyl dihydroxyphenyllactaoyl- tartaric acid	0.00 ± 0.00 a	0.21 ± 0.02 b	0.56 ± 0.07 c	0.64 ± 0.06 c	0.70 ± 0.10 c
1-O-p-coumaroylglycerol	0.00 ± 0.00 a	0.00 ± 0.00 a	0.20 ± 0.08 b	0.31 ± 0.02 b	0.40 ± 0.05 c
p-coumaroylspermidine	0.00 ± 0.00 a	0.27 ± 0.02 c	0.10 ± 0.04 b	0.00 ± 0.00 a	0.00 ± 0.00 a
di-p-coumaroylspermidine	0.30 ± 0.00 b	0.14 ± 0.03 a	0.10 ± 0.00 a	0.00 ± 0.00 a	0.00 ± 0.00 a
Feruloylquinic acid	0.09 ± 0.00 a	0.00 ± 0.00 a	0.21 ± 0.08 b	0.00 ± 0.00 a	0.00 ± 0.00 a
Flavon-3-ol (mg/100 g DM)					
(+) catechin	0.00 ± 0.00 a	0.22 ± 0.01 b	0.20 ± 0.00 b	0.28 ± 0.03 c	0.37 ± 0.04 d
procyanidin B2	0.00 ± 0.00 a	0.21 ± 0.00 b	0.71 ± 0.17 c	0.98 ± 0.00 d	1.16 ± 0.12 e
(-)epicatechin	0.00 ± 0.00 a	0.22 ± 0.01 b	0.37 ± 0.05 c	0.53 ± 0.00 d	0.92 ± 0.00 e
Dihydrochalcone (mg/100 g DM)					
phloretin-2-O-xylosylglucoside	0.00 ± 0.00 a	0.11 ± 0.00 b	0.29 ± 0.01 c	0.42 ± 0.04 d	0.69 ± 0.05 e
phloretin 2-O-glucoside (phloridzin)	0.00 ± 0.00 a	1.74 ± 0.00 b	4.02 ± 0.23 c	5.12 ± 0.05 d	6.10 ± 0.07 e

* Different letters in the row represent the statistically significant difference of average values (α = 0.05).

**Table 4:** Dietary fibre content, texture and max cut energy in wheat pasta with apple pomace.

Sample	Dietary Fibre g/100 g DM			Hardness (N)	Max Cut Energy (J)
	Insoluble Fraction	Soluble Fraction	Total Fibre		
Control *	1.37 ± 0.01 a *	2.17 ± 0.05 a	3.54 ± 0.06 a	2.08 ± 0.21 c *	3.03 ± 0.56 c
P 10%	6.26 ± 0.08 b	4.75 ± 0.03 b	11.01 ± 0.05 b	2.14 ± 0.17 cd	2.41 ± 0.38 ab
P 20%	10.88 ± 0.10 c	6.02 ± 0.04 c	16.90 ± 0.06 c	2.01 ± 0.24 c	2.50 ± 0.28 b
P 30%	15.97 ± 0.05 d	8.12 ± 0.08 d	24.08 ± 0.03 d	1.60 ± 0.26 b	2.30 ± 0.54 ab
P 50%	24.96 ± 0.07 e	11.77 ± 0.10 e	36.73 ± 0.17 e	1.44 ± 0.15 a	2.04 ± 0.45 a

* Different letters in the column represent the statistically significant difference of average values (α = 0.05).

## Data Availability

The data is included in this article.
